# Non-chemotherapy drugs inducing agranulocytosis: a disproportionality analysis based on the FAERS database

**DOI:** 10.3389/fphar.2025.1525307

**Published:** 2025-03-05

**Authors:** Shanshan Wu, Lina Huang, Jiajia Chen, Xiaochun Xie, Shaokai Huang, Xiaojie Huang

**Affiliations:** Department of Clinical Pharmacy, Jieyang People’s Hospital, Jieyang, China

**Keywords:** non-chemotherapy drug, agranulocytosis, pharmacovigilance, risk classification system, clinical monitoring

## Abstract

**Introduction:**

Non-chemotherapy drug-induced agranulocytosis (NCDIA) is a serious adverse reaction that significantly increases the risk of life-threatening infections. Although the association between certain non-chemotherapy drugs and agranulocytosis has been documented, a comprehensive analysis using a large-scale pharmacovigilance database is lacking. This study aimed to systematically identify and characterize NCDIA by analyzing adverse event reports from the FAERS database.

**Methods:**

We conducted a retrospective analysis of NCDIA reports from the FAERS database spanning from 2004 to 2024 Q1. Drugs were classified using the Anatomical Therapeutic Chemical (ATC) classification system, with chemotherapy agents (ATC code L01) excluded. The Reporting Odds Ratio (ROR) method was employed to detect potential adverse event signals. Positive signals were defined as cases with at least three reports and a lower 95% confidence interval (CI) of ROR greater than one. Time-to-event analysis was also performed to examine onset patterns across different demographic groups and drugs.

**Results:**

A total of 10,913 NCDIA reports were identified from the FAERS database. Disproportionality analysis revealed significant signals for 166 non-chemotherapy drugs related to agranulocytosis, which were systematically classified into three risk categories: known (n = 111), possible (n = 25), and new potential risks (n = 30). This classification system enables us to identify drugs with known risks, those that might pose a risk, and new risks warranting further investigation. Demographic analysis revealed that females, children (<18 years), and the elderly (≥65 years) experienced earlier onset of agranulocytosis. Drug-specific onset timing analysis provided evidence for optimizing monitoring protocols. Notably, NCDIA-associated mortality rates showed a significant decrease from 11.91% (2004–2010) to 7.28% (2021–2024) (*P* < 0.001).

**Conclusion:**

This comprehensive pharmacovigilance study not only confirmed previously known NCDIA associations but also identified new potential risk drugs. The novel risk classification system and detailed onset timing analysis provide valuable insights for clinical monitoring. The findings of earlier onset in specific populations and declining mortality trends have important implications for developing targeted surveillance strategies and improving patient safety management.

## 1 Introduction

Agranulocytosis is a critical hematological disorder marked by a drastically reduced neutrophil count, with an absolute neutrophil count below 0.5 × 10^9^/L (Andrès and Maloisel, 2008; Lorenzo-Villalba et al., 2020). This condition significantly elevates the risk of severe infections, posing a serious threat to patient health (Andres and Mourot-Cottet, 2017). The majority of agranulocytosis cases, approximately 70%–97%, are induced by medications (Garbe, 2007; Rattay and Benndorf, 2021). These drug-induced cases can be classified into two primary categories based on their mechanisms: chemotherapy drug-induced agranulocytosis (CDIA) and non-chemotherapy drug-induced agranulocytosis (NCDIA) (Andres et al., 2011; Andres et al., 2019).

Chemotherapy drugs are a well-recognized cause of agranulocytosis due to their inherent mechanism of action, which involves suppressing bone marrow function by targeting rapidly dividing cells. This suppression often results in severe neutropenia, serving as a dose-limiting toxicity (Lyman et al., 2014). Managing CDIA often involves adjusting drug doses, delaying treatment, and using granulocyte-colony stimulating factors (G-CSF) to stimulate white blood cell production and reduce the risk of severe infections (Lalami and Klastersky, 2017).

In contrast, non-chemotherapy drugs encompass a wide array of medications from various therapeutic classes not typically associated with cancer treatment. These drugs can induce agranulocytosis through immunological or cytotoxic mechanisms (Andres and Mourot-Cottet, 2017). Unlike CDIA, where the risk is more clearly linked to dose and duration of treatment, NCDIA often exhibits no clear dose dependence, leading to its idiosyncratic nature. Additionally, some drugs require metabolism into active products to cause agranulocytosis, with certain cases influenced by genetic polymorphisms that produce active metabolites only under specific disease conditions (Rattay and Benndorf, 2021). This contributes to the relative rarity and unpredictability of such adverse reactions, presenting unique challenges in clinical practice. Therefore, early identification and prompt management of NCDIA are crucial to preventing severe infectious complications. Raising awareness and educating healthcare providers about the signs and symptoms of agranulocytosis, regularly monitoring blood counts in high-risk patients, and thoroughly reviewing patient history for any previous drug-induced reactions can help mitigate these risks and enhance patient safety (Curtis, 2017).

Recently, we developed the first machine learning model to predict NCDIA toxicity, which integrates molecular descriptors and chemical structure information to provide a valuable tool for assessing agranulocytosis risk in drug development and clinical monitoring (Huang et al., 2024). However, to the best of our knowledge, although the association between certain non-chemotherapy drugs and agranulocytosis has been documented, a comprehensive analysis using a large-scale pharmacovigilance database is lacking. Understanding the characteristics and risk patterns of NCDIA from a pharmacovigilance perspective is crucial for developing personalized monitoring strategies and optimizing patient safety management in clinical practice. This study aims to leverage the FAERS dataset to deepen and expand the existing knowledge on NCDIA.

## 2 Materials and methods

### 2.1 Data source

The data for this study were sourced from the FAERS database (available at https://fis.fda.gov/extensions/FPD-QDE-FAERS/FPD-QDE-FAERS.html, accessed on 15 June 2024), covering a 20-year period from 2004 to the first quarter of 2024 (2024 Q1).

### 2.2 Target adverse event and drug classification criteria

To ensure a rigorous pharmacovigilance analysis of non-chemotherapy drug-induced agranulocytosis, we established specific criteria for both adverse event identification and drug classification. For adverse event selection, we focused exclusively on the Preferred Term (PT) “Agranulocytosis” according to MedDRA version 27.0. While the Standardized MedDRA Query (SMQ) of agranulocytosis could provide a broader coverage including related conditions such as aplastic anemia, bone marrow failure, and febrile neutropenia, this focused approach was chosen to avoid potential confounding from conditions that might have different underlying mechanisms or clinical manifestations, thereby enabling a more specific analysis of drug-induced agranulocytosis cases, though it may result in a more conservative estimate of case numbers. For drug classification, we employed the Anatomical Therapeutic Chemical (ATC) classification system to systematically analyze agranulocytosis adverse events (AEs) induced by non-chemotherapy drugs. Drugs coded under “L01: antineoplastic agents” were classified as chemotherapy drugs, while those with different ATC codes were categorized as non-chemotherapy drugs, allowing for a focused evaluation of their association with agranulocytosis.

### 2.3 Data extraction and processing

Data cleaning was performed in strict accordance with the FDA’s official guidelines, as outlined in the flowchart in Supplementary Figure S1. To remove duplicate records, we selected the PRIMARYID, CASEID, and FDA_DT fields from the DEMO table. The data were sorted by CASEID, FDA_DT, and PRIMARYID. For reports with the same CASEID, we retained the one with the latest FDA_DT value. If both CASEID and FDA_DT were identical, we retained the report with the highest PRIMARYID value. Since 2019, the delete file included with each quarter’s data indicates which reports need to be removed from the database. Additionally, only data related to the “primary suspect drug” were extracted and utilized for constructing the data analysis table. After data cleaning and integration, reports related to NCDIA were extracted.

Data extraction, analysis, and visualization were performed using SAS 9.4, R software (version 4.2.1), WPS Office, and Python (version 3.9.12).

### 2.4 AE signal detection and statistical analysis

In this study, we employed the Reporting Odds Ratio (ROR) method, a well-established disproportionality analysis technique in pharmacovigilance research, to detect potential AE signals (Sakaeda et al., 2013). The calculation formula for ROR and its corresponding 95% confidence interval (CI) are presented in Supplementary Table S1. A drug is considered to have a potential risk of inducing agranulocytosis if there are three or more reports of the target AE and the lower limit of the 95% CI for the ROR exceeds one. For the disproportionality analysis, the reference group consisted of all adverse event reports in FAERS after excluding reports related to chemotherapy drugs (ATC code L01) and duplicate reports, as detailed in the data processing flowchart (Supplementary Figure S1).

For statistical analysis, continuous variables were presented as median and interquartile range (IQR), and categorical variables as counts and percentages. The chi-square test was used to assess differences in categorical variables, with Bonferroni correction for pairwise mortality rate comparisons across time periods. The Mann-Whitney U test was employed for two-group comparisons of continuous variables, while the Kruskal-Wallis test followed by Dunn’s test with Bonferroni correction was used for multiple group comparisons. For multiple comparisons, P values were adjusted using Bonferroni correction, and the adjusted P values were reported. For all analyses, a two-sided P value <0.05 was considered statistically significant.

### 2.5 Classification of NCDIA risk

To systematically evaluate the agranulocytosis risk of identified drugs, we developed a three-tier classification system. Drugs were categorized based on literature review of Micromedex (assessed on 14 July 2024), the SIDER database (Kuhn et al., 2016) (assessed on 15 July 2024), and relevant published reviews (Andersohn et al., 2007; Garbe, 2007; Andres and Mourot-Cottet, 2017; Andrès and Maloisel, 2008; Coates, 2024). Drugs were classified as: (1) “Known NCDIA”: drugs with documented “agranulocytosis” as an adverse effect in any of these sources; (2) “Possible NCDIA”: drugs without documented agranulocytosis but with records of other hematologic adverse reactions in Micromedex involving decreased white blood cell or neutrophil counts (e.g., febrile neutropenia, neutropenia, leukopenia, decreased white blood cell count, aplastic anemia); (3) “New Potential NCDIA”: drugs without documented hematologic adverse effects.

## 3 Results

### 3.1 Basic information on patient characteristics

**FIGURE 1:**
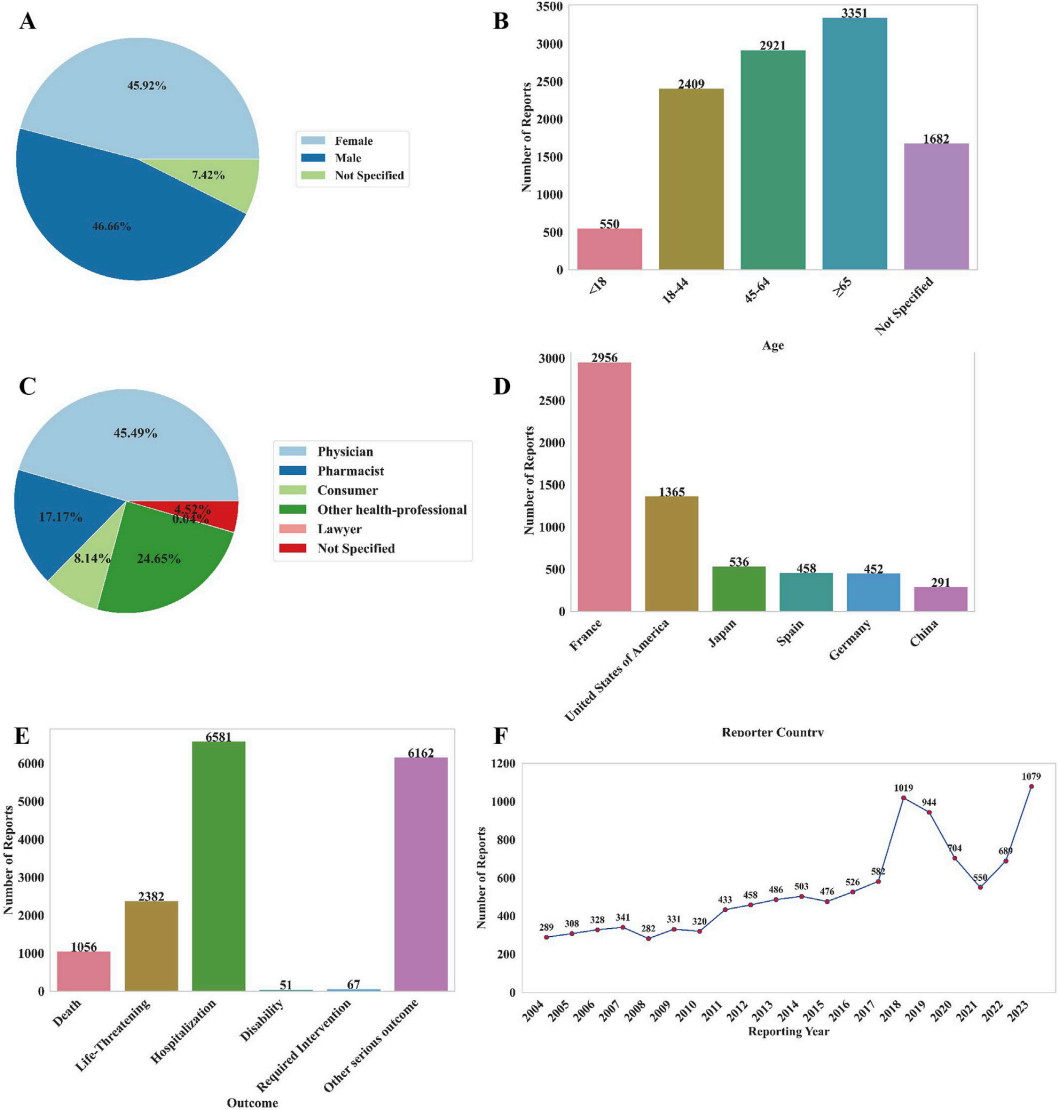
Basic information of AE reports related to NCDIA from 2004 to 2024. **(A)** Distribution of patient gender. **(B)** Distribution of patient age. **(C)** Distribution of reporters’ occupations. **(D)** Top six countries with the highest number of reports. **(E)** Outcome distribution of adverse reactions in patients. **(F)** Annual number of AE reports.

From 2004 to 2024 Q1, the FAERS database recorded a total of 44472692 AE reports related to non-chemotherapy drugs. Among these, 10,913 reports involving 10,913 patients were associated with NCDIA. The basic characteristics of patients with NCDIA are provided in Supplementary Table S2. As illustrated in Figure 1A, the reporting ratio of NCDIA is similar between genders, with 45.92% of reports involving females and 46.66% involving males (7.42% unknown), indicating no significant gender bias in NCDIA occurrence (*P* = 0.420). The median age of patients was 57 years (IQR: 39–71), with elderly patients (age ≥65, n = 3351, 30.71%) showing a significantly higher reporting proportion compared to other age groups (all *P* < 0.001, Bonferroni-adjusted) (Figure 1B). The majority of reports were submitted by physicians, accounting for 45.49% of NCDIA cases (Figure 1C). Regarding geographical distribution, France contributed the highest number of reports, making up 27.09% (n = 2956) of the total (Figure 1D). In terms of patient outcomes, NCDIA resulted in serious adverse outcomes, with 21.83% (n = 2382) of cases being life-threatening and 9.68% (n = 1056) of cases resulting in patient deaths (Figure 1E). As shown in Figure 1F, the number of NCDIA reports surged in 2023, reaching a historical high of 1079 cases.

### 3.2 Non-chemotherapy drugs and drug classes associated with agranulocytosis

**FIGURE 2:**
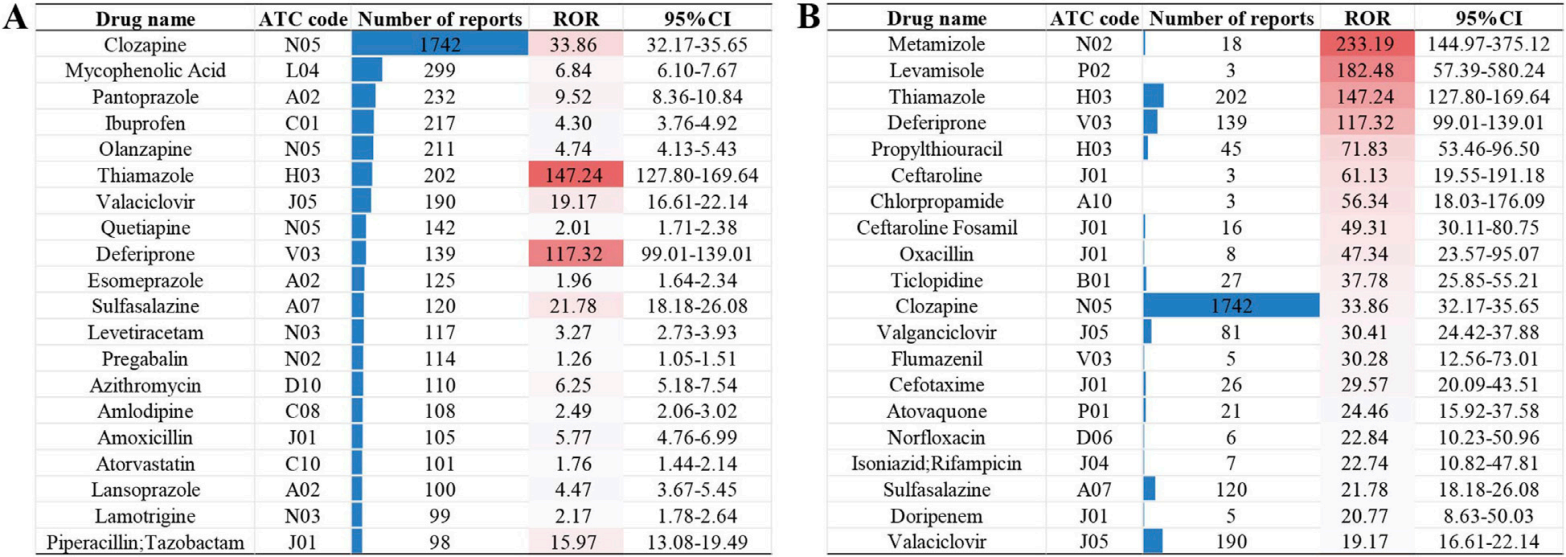
The top 20 drugs with the highest number of target reports **(A)** and highest ROR values **(B)**.

Among the 10,913 reports associated with NCDIA, a total of 166 non-chemotherapy drugs were identified with a positive AE signal, as detailed in Supplementary Table S3. Figure 2 provides an overview of the top 20 drugs with the highest number of target reports (Figure 2A) and highest ROR values (Figure 2B). The top five non-chemotherapy drugs, based on the number of reports, are Clozapine (n = 1742, ROR = 33.86), followed by Mycophenolic Acid (n = 299, ROR = 6.84), Pantoprazole (n = 232, ROR = 9.52), Ibuprofen (n = 217, ROR = 4.30), and Olanzapine (n = 211, ROR = 4.74). When considering ROR values, Metamizole has the highest ROR (n = 18, ROR = 233.19), followed by Levamisole (n = 3, ROR = 182.48), Thiamazole (n = 202, ROR = 147.24), Deferiprone (n = 139, ROR = 117.32), and Propylthiouracil (n = 45, ROR = 71.83). Overall, a higher ROR value suggests a greater risk of the drug causing agranulocytosis, underscoring the need for heightened vigilance regarding these high-risk drugs in clinical practice.

**FIGURE 3:**
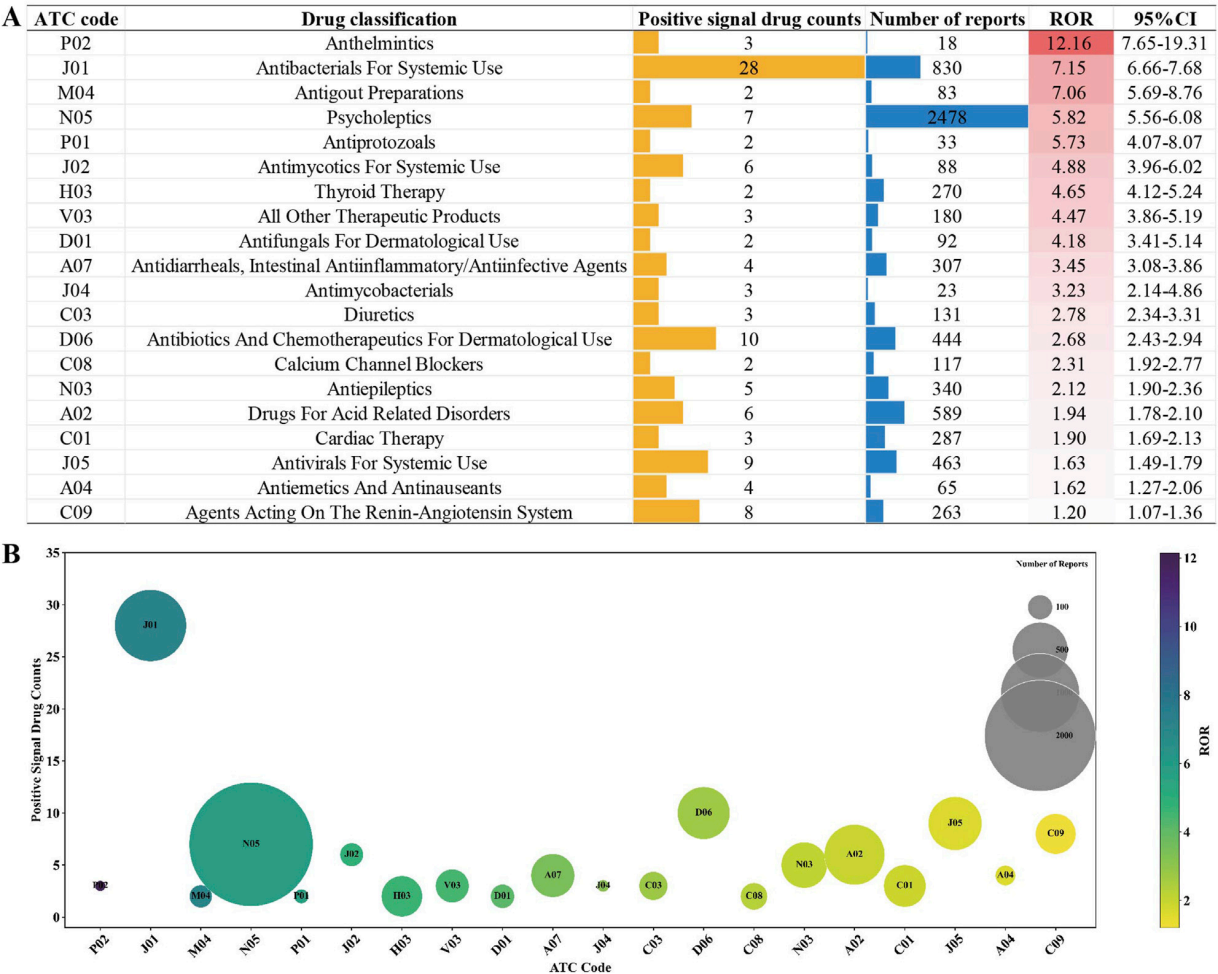
Non-chemotherapy drug classes with positive AE signals based on ATC code: **(A)** Drug classes ranked by ROR. **(B)** Bubble chart illustration: This chart visually represents the number of drugs with positive AE signals across different drug classes (vertical axis), the total number of reports for each drug class (bubble size), and their respective ROR values (color intensity).

In addition, we assessed the risk of agranulocytosis across different drug classes based on the second level of the ATC code. A total of 20 drug classes were identified as high-risk for agranulocytosis, encompassing 112 drugs with positive AE signals. This means that the remaining 54 drugs with positive AE signals belong to classes not considered high-risk for agranulocytosis. The results for non-chemotherapy drug classes with positive AE signals, ranked by ROR, are presented in Figure 3A and further illustrated in the bubble chart in Figure 3B. The class with the highest risk of agranulocytosis is P02 (Anthelmintics), with an ROR of 12.16 and three drugs identified with positive signals (Albendazole, Mebendazole, and Levamisole). Other classes with significant ROR values include J01 (Antibacterials for Systemic Use), M04 (Antigout Preparations), N05 (Psycholeptics), and P01 (Antiprotozoals), each with an ROR value exceeding 5. This indicates that these drug classes are associated with a higher risk of agranulocytosis.

### 3.3 Identifying non-chemotherapy drugs with new potential agranulocytosis risk

**FIGURE 4:**
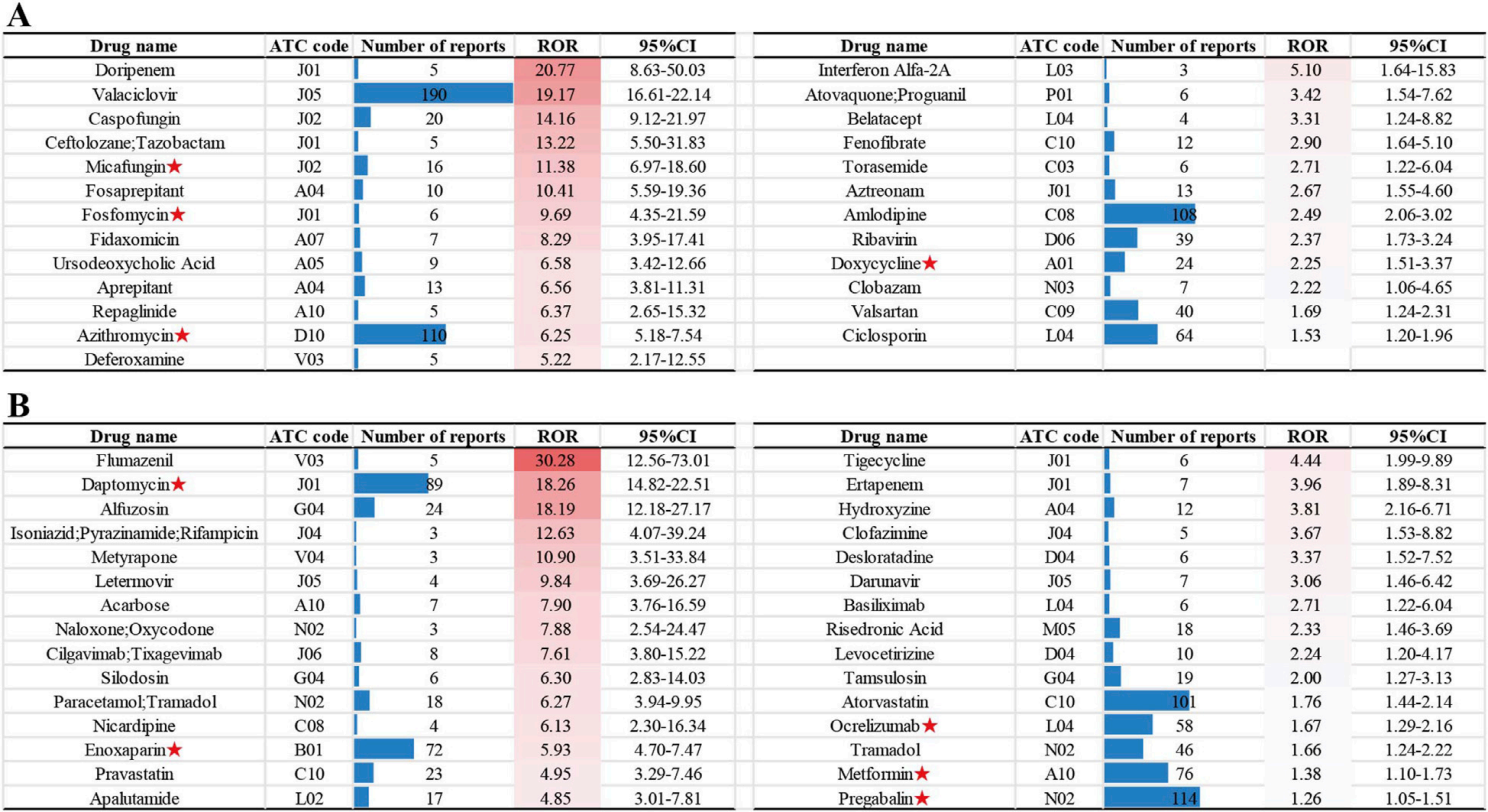
Non-chemotherapy drugs with new potential agranulocytosis risk: **(A)** “Possible NCDIA” drugs ranked by ROR values. **(B)** “New Potential NCDIA” drugs ranked by ROR values. Drugs with agranulocytosis case reports are marked with red asterisks.

To better understand and characterize the risk profile of drugs associated with NCDIA, we analyzed the 166 drugs with positive signals using a novel three-tier classification system (Known NCDIA, Possible NCDIA, and New Potential NCDIA) as introduced in the Methods section. This classification system enables us to identify drugs with known risks, those that might pose a risk, and new risks warranting further investigation. Among the drugs analyzed, 111 were categorized as “Known NCDIA”, 25 as “Possible NCDIA”, and 30 as “New Potential NCDIA.” The categories of 166 non-chemotherapy drugs are presented in Supplementary Table S3, with the “Possible NCDIA” drugs ranked by their ROR values in Figure 4A, and the “New Potential NCDIA” drugs shown in Figure 4B.

Additionally, we conducted a search on PubMed (assessed on 15 July 2024) for agranulocytosis case reports related to drugs in the “Possible NCDIA” and “New Potential NCDIA” categories. From the literature review, we identified four drugs in the “Possible NCDIA” category with agranulocytosis case reports: Micafungin (Ameri et al., 2019), Fosfomycin (Matusik et al., 2023), Azithromycin (Kajiguchi and Ohno, 2009), and Doxycycline (Parent-Massin et al., 1993), and five drugs in the “New Potential NCDIA” category: Daptomycin (Leyra et al., 2016), Enoxaparin (Medrano-Casique et al., 2016), Ocrelizumab (Auer et al., 2020), Metformin (Laporte et al., 2008), and Pregabalin (Kino et al., 2014). These drugs with agranulocytosis case reports are marked with red asterisks in Figure 4.

### 3.4 Time to event analysis of agranulocytosis for non-chemotherapy drugs

Among the 10,913 NCDIA reports, 5046 cases included recorded time-to-event data for agranulocytosis. Table 1 presents the analysis of the time to onset of NCDIA, categorized by gender and age groups.

**TABLE 1:** Time to event of agranulocytosis for non-chemotherapy drugs by gender and age groups.

Category	Number of cases	Median time (IQR) (days)	Statistical test	*P*-value
Overall	5046	22 (7–58)	—	—
Gender			Mann-Whitney U test	0.001*
Female	2399	20 (7–55)	—	—
Male	2445	23 (7–62)	—	—
Unknown	202	—	—	—
Age group			Kruskal-Wallis test	<0.001*
<18	268	18 (4–50)	—	—
18–44	1039	23 (6–93)	—	—
45–64	1454	25 (8–80)	—	—
≥65	1824	19 (7–43)	—	-
Unknown	461	—	—	—
Comparison of time to onset among age groups	—	—	Dunn’s test with Bonferroni correction	—
<18 vs. 18–44	—	—	—	<0.001*
<18 vs. 45–64	—	—	—	<0.001*
<18 vs. ≥ 65	—	—	—	0.847
18–44 vs. 45–64	—	—	—	0.901
18–44 vs. ≥ 65	—	—	—	<0.001*
45–64 vs. ≥ 65	—	—	—	<0.001*

Statistical significance is marked by asterisks (*).

**FIGURE 5:**
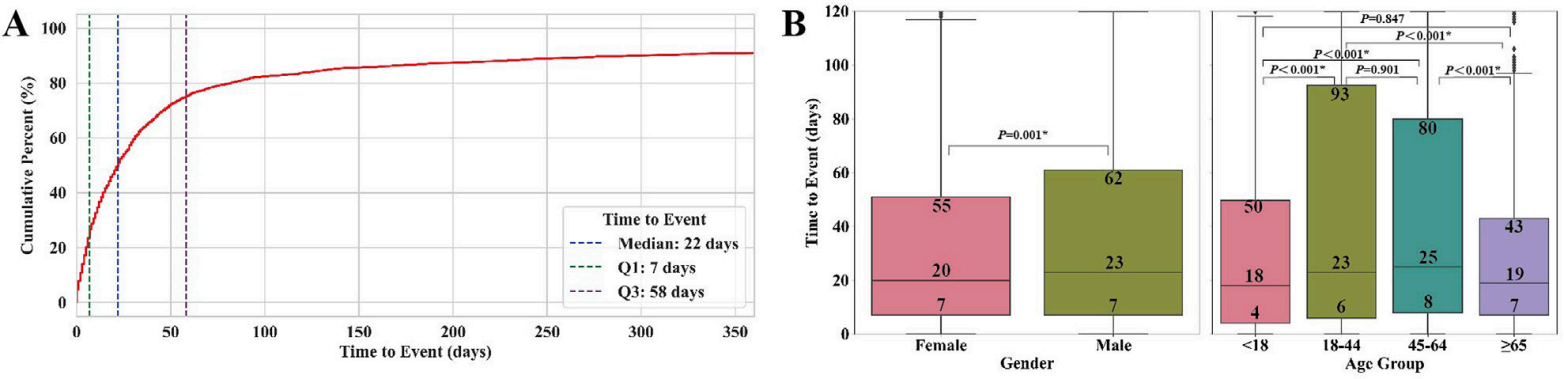
Time to event of agranulocytosis for non-chemotherapy drugs: **(A)** Cumulative percentage curve of time to onset. **(B)** Box plots of time to onset by gender and age groups. * indicates statistical significance between two groups.

The overall median time to event for NCDIA was 22 days (IQR, 7–58), as shown in the cumulative percentage curve (Figure 5A). The box plots depicting time to event by gender and age groups are presented in Figure 5B. Notable gender differences were observed, with females showing earlier onset (median: 20 days, IQR: 7–55) compared to males (median: 23 days, IQR: 7–62; *P* = 0.001). Age group analysis showed marked variability in the time to onset of agranulocytosis. Patients under 18 years had a median onset time of 18 days (IQR, 4–50), those aged 18–44 years had a median of 23 days (IQR, 6–93), patients aged 45–64 years had a median of 25 days (IQR, 8–80), and those aged 65 years or older had a median of 19 days (IQR, 7–43). The Kruskal-Wallis test indicated significant differences among these age groups (*P* < 0.001). Further analysis using Dunn’s test with Bonferroni correction identified specific significant differences between several age groups, particularly highlighting that patients under 18 and those 65 years and older had earlier onset times compared to other groups (<18 vs. 18–44: *P* < 0.001; <18 vs. 45–64: *P* < 0.001; 18–44 vs. ≥ 65: *P* < 0.001; 45–64 vs. ≥ 65: *P* < 0.001). These results suggest that both gender and age significantly impact the onset time of agranulocytosis in patients treated with non-chemotherapy drugs. The earlier onset in females and in age groups (<18 and ≥65 years) may reflect underlying biological differences or variations in drug metabolism or immune response. Clinicians should consider these factors when monitoring patients for agranulocytosis, tailoring surveillance and intervention strategies to effectively mitigate risks.

Drug-specific analysis of cases with ≥20 reports revealed substantial variations in onset timing (Supplementary Table S4). Azithromycin showed the most rapid onset (median: 4 days, IQR: 2–6), while Ocrelizumab demonstrated the longest latency (median: 176 days, IQR: 86–557). Similar variations were observed among drug classes (Supplementary Table S5), with C01 (Cardiac Therapy) showing the shortest onset (median: 6 days, IQR: 2–21) and V03 (All Other Therapeutic Products) the longest (median: 73 days, IQR: 18–186). The significant variation observed in the time to onset among non-chemotherapy drugs underscores the importance of understanding these specific onset times for individual drugs and drug classes. Such insights can enhance the prediction and management of NCDIA.

### 3.5 NCDIA followed by death

**FIGURE 6:**
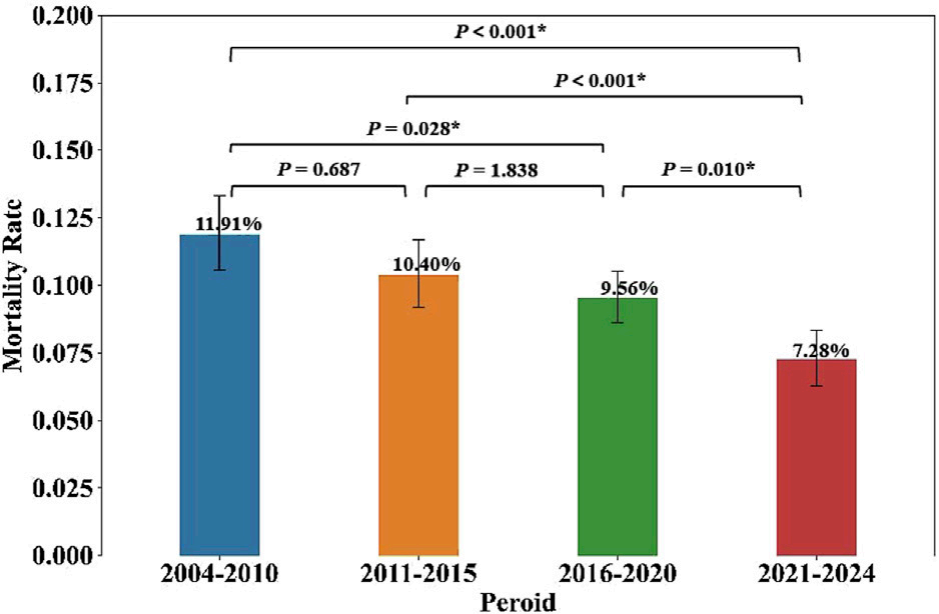
Mortality rates of NCDIA across four time periods. Statistical significance (*P* < 0.05) is marked by asterisks (*), with *P*-values shown for comparisons between each period.

During the study period, the FDA received a total of 1056 reports (9.68%) of agranulocytosis cases followed by death attributed to non-chemotherapy drugs. A summary of the death and non-death cases of NCDIA is provided in Table 2. The median age of NCDIA death patients was 65 years (IQR, 50–77), and 48.67% of these patients were female. Statistical tests showed no significant difference in gender distribution and time to onset of agranulocytosis between death and non-death cases (*P* = 0.070 and *P* = 0.126, respectively). However, there was a significant difference in age between the two groups, with death cases having a higher median age compared to non-death cases (*P* < 0.001). This suggests that age is a significant factor in the mortality of agranulocytosis patients. Additionally, the study found a decreasing trend in the mortality rate of NCDIA from 2004 to 2024. The 20-year period was divided into four intervals: 2004–2010, 2011–2015, 2016–2020, and 2021–2024. As shown in Figure 6, the mortality rate of NCDIA decreased significantly, from 11.91% (95% CI: 10.59–13.34) in 2004–2010 to 7.28% (95% CI: 6.31–8.35) in 2021–2024 (*P* < 0.001).

**TABLE 2:** Summary of death and non-death cases of NCDIA.

Category	Death cases (n = 1056)	Non-death cases (n = 9857)	Statistical test	*P*-value
Gender			Chi-squared test	0.070
Female	514 (48.67%)	4497 (45.62%)	—	—
Male	467 (44.22%)	4625 (46.92%)	—	—
Unknown	75 (7.10%)	735 (7.46%)	—	—
Age			Mann-Whitney U test	<0.001*
Median years (IQR)	65 (50–77)	55 (38–70)	—	—
Missing cases	174	1508	—	—
Time to event			Mann-Whitney U test	0.126
Median days (IQR)	28 (8–58)	21 (7–58)	—	—
Missing cases	595	5272	—	—
Year period			Chi-squared test	<0.001*
2004–2010	262 (11.91%)	1937 (88.09%)	—	—
2011–2015	245 (10.40%)	2111 (89.60%)	—	—
2016–2020	361 (9.56%)	3414 (90.44%)	—	—
2021–2024	188 (7.28%)	2395 (92.72%)	—	—
Comparison of mortality rate			Pairwise Chi-square tests with Bonferroni correction	
2004–2010 vs. 2011–2015	—	—	—	0.687
2004–2010 vs. 2016–2020	-	-	-	0.028*
2004–2010 vs. 2021–2024	—	—	—	<0.001*
2011–2015 vs. 2016–2020	—	—	—	1.838
2011–2015 vs. 2021–2024	—	—	—	<0.001*
2016–2020 vs. 2021–2024	—	—	—	0.010*

Statistical significance is marked by asterisks (*).

**FIGURE 7:**
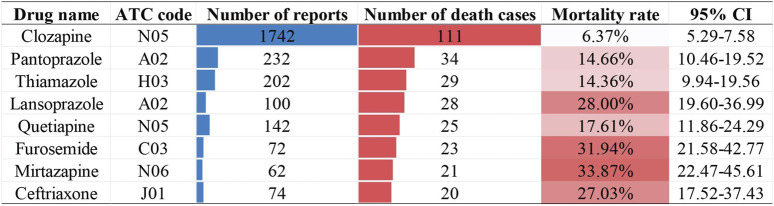
Non-chemotherapy drugs with 20 or more reports of NCDIA followed by death.

Drug-specific mortality analysis identified eight drugs associated with ≥20 death reports (Figure 7). While Clozapine had the highest number of death cases, its mortality rate (6.37%, 95% CI: 5.29–7.58) was the lowest among these drugs. Notably, Mirtazapine and Furosemide demonstrated the highest mortality rates (33.87%, 95% CI: 22.47%–45.61% and 31.94%, 95% CI: 21.58–42.77, respectively).

## 4 Discussion

NCDIA is a severe and life-threatening adverse drug reaction, characterized by its complex mechanisms and idiosyncratic nature. Despite increased research and heightened awareness in recent years, predicting and managing NCDIA remains challenging. While previous studies have documented individual cases and small-scale analyses, no comprehensive investigation has systematically evaluated NCDIA using a large-scale pharmacovigilance database. Our study addresses this gap by analyzing NCDIA reports from the FAERS database over a 20-year period, providing novel insights into its characteristics and identifying previously unrecognized risk drugs.

Firstly, gender and age play distinct roles in the occurrence and development of NCDIA. Our study indicates no significant difference in NCDIA reporting rates between genders (Supplementary Table S2). Although previous research suggested that females report NCDIA far more frequently than males,our analysis did not reveal a gender bias (Maloisel et al., 2003; Lorenzo-Villalba et al., 2020; Andres et al., 2017). Regarding age distribution, patients aged ≥ 65 years reported significantly more NCDIA cases than other age groups (Supplementary Table S2; Figure 1B). This finding aligns with previous studies identifying elderly patients (≥ 65 years) as a major risk factor for NCDIA (Lorenzo-Villalba et al., 2020). Elderly patients are more susceptible to NCDIA due to factors such as declining liver and kidney function, multiple disease comorbidities, and the use of various medications (Lorenzo-Villalba et al., 2020). This underscores the need for clinicians to exercise increased caution and implement enhanced monitoring and preventive measures when prescribing high-risk non-chemotherapy drugs to elderly patients. Additionally, our study systematically analyzed the timing of NCDIA onset and found significant differences across gender and age groups (Table 1; Figure 5B). Females tend to develop agranulocytosis earlier than males, and patients under 18 and over 65 years old tend to develop it earlier than other age groups. Previous studies have shown that adverse drug reactions vary by gender and age (Alomar, 2014; Gandhi et al., 2004; Soldin and Mattison, 2009; Corsonello et al., 2010). The observed gender differences in NCDIA onset may be due to biological and physiological factors such as variations in drug metabolism enzyme activity and hormone levels, which could affect the timing of drug-induced agranulocytosis (Gandhi et al., 2004; Soldin and Mattison, 2009). Age differences are attributed to the distinct metabolic and immune systems in elderly and pediatric patients compared to young and middle-aged adults (Corsonello et al., 2010; Simon et al., 2015; Alomar, 2014). Previous epidemiological studies on NCDIA did not report differences in the timing of agranulocytosis onset by gender and age. Therefore, our findings provide valuable insights for clinicians and pharmacists to develop better monitoring and intervention strategies, especially for high-risk populations.

Secondly, this study identified a variety of drugs with potential high risk for agranulocytosis. From 10,913 reports of NCDIA, we identified 166 drugs with positive signals (Supplementary Table S3). After an in-depth database and literature review, we established a classification system for NCDIA: 111 drugs were categorized as “Known NCDIA”, 25 as “Possible NCDIA”, and 30 as “New Potential NCDIA”. In clinical practice, it is crucial to be particularly vigilant regarding drugs categorized as “Possible NCDIA” and “New Potential NCDIA” (Figure 4). Although these drugs have not been previously identified as causing agranulocytosis in their labeling, those classified as “Possible NCDIA” have shown adverse reactions related to neutropenia or leukopenia. If not monitored and managed, these reactions could potentially progress to agranulocytosis. Conversely, “New Potential NCDIA” drugs have no documented adverse effects related to agranulocytosis or granulocytopenia in their labeling. However, our research indicates a risk signal for agranulocytosis with these drugs, and some already have associated case reports of agranulocytosis. For instance, further literature review revealed case reports of agranulocytosis associated with four “Possible NCDIA” drugs: Micafungin (Ameri et al., 2019), Fosfomycin (Matusik et al., 2023), Azithromycin (Kajiguchi and Ohno, 2009), and Doxycycline (Parent-Massin et al., 1993); as well as with five “New Potential NCDIA” drugs: Daptomycin (Leyra et al., 2016), Enoxaparin (Medrano-Casique et al., 2016), Ocrelizumab (Auer et al., 2020), Metformin (Laporte et al., 2008), and Pregabalin (Kino et al., 2014). Notably, among these nine drugs, only Doxycycline was included in a systematic review of case reports in 2007 (Andersohn et al., 2007). The other eight drugs had case reports published after 2008. Due to the low incidence and idiosyncratic nature of this adverse drug reaction, many drugs that cause agranulocytosis are identified through post-marketing surveillance rather than during clinical trials, as the trials often have strict inclusion and exclusion criteria (Rattay and Benndorf, 2021). Therefore, signal mining of adverse drug reactions in the FAERS database, along with case report analysis, is crucial for understanding the characteristics of agranulocytosis caused by specific drugs.

Furthermore, our study provides comprehensive insights into NCDIA onset patterns. Beyond demographic analyses, we characterized drug-specific and drug class-specific onset timing (Supplementary Tables S4, S5), revealing substantial variations that have important implications for clinical monitoring. For instance, onset times ranged from 4 days (Azithromycin) to 176 days (Ocrelizumab) among individual drugs, and from 6 days (C01: Cardiac Therapy) to 73 days (V03: All Other Therapeutic Products) among drug classes. This temporal characterization addresses a significant knowledge gap, as comprehensive onset timing data for NCDIA has been largely absent from the literature. Such information is crucial for clinical practice, enabling: (1) Evidence-based assessment of potential drug-induced agranulocytosis cases; (2) Optimization of blood count monitoring frequencies; (3) Development of drug-specific surveillance protocols. The clinical utility of such timing data is well illustrated by Thiamazole, where we observed a median onset time of 42 days (IQR: 30–66 days). This aligns with findings from a Japanese study showing that biweekly blood count monitoring during the first 2 months of Thiamazole therapy can identify 78% of neutropenia cases before progression to agranulocytosis (Nakamura et al., 2013). This example demonstrates how onset timing data can inform practical monitoring strategies to prevent severe complications.

Finally, our mortality analysis revealed important patterns and trends in NCDIA outcomes. Agranulocytosis, characterized by a severe deficiency of neutrophils, can precipitate life-threatening infections and, in extreme cases, lead to patient death. European research reports indicate that over the past 2 decades, the mortality rate associated with NCDIA has ranged between 10% and 16% (Andres and Mourot-Cottet, 2017). High-risk factors for mortality in patients with agranulocytosis include advanced age (over 65 years), renal insufficiency (serum creatinine level >120 μmol/L), and the presence of bacteremia or shock (Andrès and Maloisel, 2008; Andres et al., 2011). Our research corroborates these findings, showing a mortality rate of 9.68% for NCDIA based on reports from 2004 to 2024. The median age of deceased cases was 65 years (IQR 50–77), significantly higher than the median age of non-fatal cases, which was 55 years (IQR 38–70). Furthermore, we identified eight drugs with more than twenty reported fatal cases of agranulocytosis (Figure 7), among which Clozapine had the highest number of fatal cases but the lowest mortality rate at 6.37%. This may be attributed to increased awareness and preventive measures against clozapine-induced agranulocytosis (Mijovic and MacCabe, 2020). In the United States, the FDA mandates regular monitoring and reporting of neutrophil counts for all patients on Clozapine, facilitating safe use of the drug while mitigating mortality associated with clozapine-induced agranulocytosis. Between 1990 and 1994 in the United States, the incidence of clozapine-induced agranulocytosis decreased from 1% to 2% in pre-marketing studies to 0.38% post-marketing, with the mortality rate declining from an estimated 15% to approximately 3.1% (Honigfeld, 1996). Despite the life-threatening potential of NCDIA, our study indicates a positive trend, with the mortality rate declining from 11.91% during 2004–2010 to 7.28% during 2021–2024 (Table 2; Figure 6). This downward trend may be attributed to improvements in intensive care treatments, the availability of effective broad-spectrum antibiotics, heightened physician vigilance regarding drug-induced agranulocytosis, prompt cessation of suspected drugs, and the proactive use of G-CSF or GM-CSF in patients with severe agranulocytosis (Andersohn et al., 2007). These measures collectively contribute to improving the therapeutic outcomes for patients with agranulocytosis and reducing mortality rates.

However, several limitations of this study should be acknowledged. First, our case identification relied solely on the Preferred Term (PT) “Agranulocytosis” rather than using the SMQ of agranulocytosis that include related conditions such as aplastic anemia, bone marrow failure, febrile neutropenia, and neutropenic sepsis. While this focused approach ensures high specificity for agranulocytosis cases and avoids potential confounding from conditions with different pathophysiological mechanisms or clinical manifestations, it likely results in under-detection of some relevant cases. This conservative case identification strategy means our findings might underestimate the true scope of NCDIA conditions. A second fundamental limitation stems from the inherent constraints of the FAERS database regarding laboratory data verification. Although agranulocytosis is strictly defined as an absolute neutrophil count below 0.5 × 10^9^/L, FAERS does not contain laboratory data to confirm these values. Consequently, an unknown proportion of the reported cases may not meet the strict laboratory definition of agranulocytosis. However, given that agranulocytosis is a severe condition requiring immediate medical attention, most reports likely originate from healthcare professionals who have made the diagnosis based on laboratory findings, even though these values are not captured in the database. Additionally, like other spontaneous reporting systems, the FAERS database has limitations such as reporting bias, incomplete information, and the inability to establish true causality (Sakaeda et al., 2013; Ismail and Akram, 2022). These limitations necessitate cautious interpretation of our findings. Future pharmacovigilance studies could be strengthened by: (1) employing the SMQ of agranulocytosis for more comprehensive case identification, (2) linking with electronic health records to validate laboratory criteria, and (3) conducting prospective studies to better establish causality between specific drugs and agranulocytosis.

## 5 Conclusion

In this comprehensive pharmacovigilance study of NCDIA spanning 2 decades (2004–2024), we have made several significant findings with important clinical implications. First, we identified 166 drugs associated with NCDIA, which were then systematically categorized using our novel three-tier classification system, revealing 30 previously unrecognized potential risk drugs and providing clinicians with an updated framework for risk assessment. Second, the analysis revealed distinct gender and age-related patterns in NCDIA onset, with females and specific age groups (<18 and ≥65 years) showing earlier onset, emphasizing the need for more targeted monitoring strategies in these populations. The drug-specific onset timing analysis offers valuable guidance for optimizing blood count monitoring protocols, particularly for high-risk medications. Importantly, the observed decline in NCDIA-associated mortality rates from 11.91% to 7.28% over the study period suggests improving clinical management strategies, though continued vigilance remains essential. These findings have direct implications for clinical practice, supporting the development of more personalized monitoring approaches and potentially improving patient safety in non-chemotherapy drug treatment. Future pharmacovigilance studies and clinical investigations should focus on validating the newly identified potential risk drugs and further refining monitoring strategies for specific patient populations. Our findings provide a foundation for evidence-based decision-making in NCDIA risk management and patient care.

## Data Availability

The original contributions presented in the study are included in the article/Supplementary Material, further inquiries can be directed to the corresponding author.
