# Supplementary figures and images for: Non-chemotherapy drugs inducing agranulocytosis: a disproportionality analysis based on the FAERS database
Source: Front Pharmacol. 2025 Mar 5;16:1525307. doi: 10.3389/fphar.2025.1525307 (PMC11920160; doi:10.3389/fphar.2025.1525307)

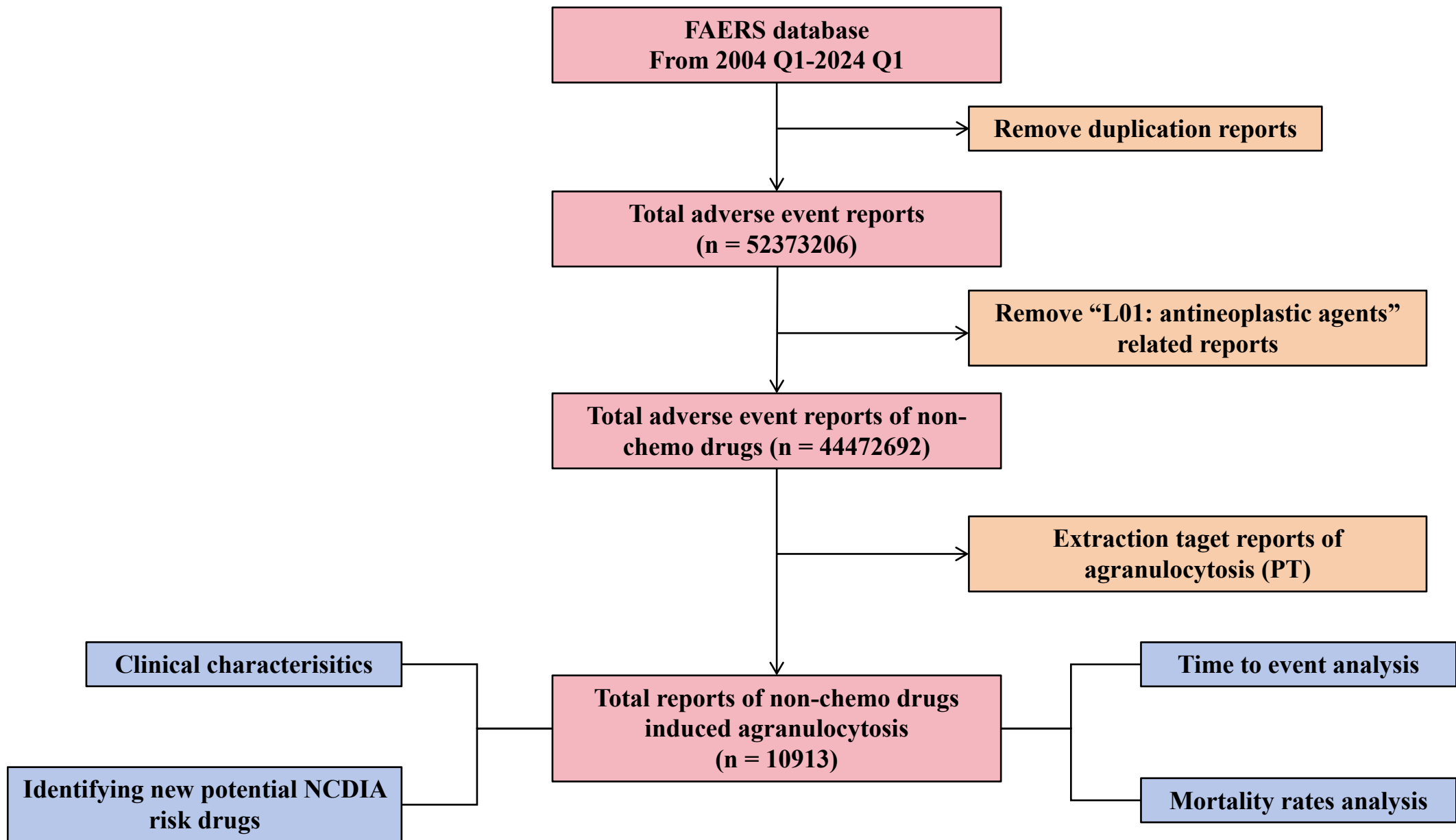

**Figure S1.** The flowchart for data extraction and processing.

Supplement: Supplementary file 2 [file Image1.pdf]
